# Phase II Study of Bortezomib as a Single Agent in Patients with Previously Untreated or Relapsed/Refractory Acute Myeloid Leukemia Ineligible for Intensive Therapy

**DOI:** 10.1155/2013/705714

**Published:** 2013-04-28

**Authors:** Chiara Sarlo, Francesco Buccisano, Luca Maurillo, Mariagiovanna Cefalo, Luigi Di Caprio, Laura Cicconi, Concetta Ditto, Licia Ottaviani, Ambra Di Veroli, Maria Ilaria Del Principe, Maria Assunta Grasso, Daniela Nasso, Giovanna De Santis, Sergio Amadori, Adriano Venditti

**Affiliations:** Department of Hematology, University of Rome Tor Vergata, Viale Oxford 81, 00133 Rome, Italy

## Abstract

We explored the safety and efficacy of bortezomib given as single agent in patients with untreated or relapsed/refractory acute myeloid leukemia (AML), unfit for conventional chemotherapy. Fourteen patients were treated with bortezomib 1.5 mg/m^2^ administered twice weekly for two weeks, every 3 weeks. Median age was 70 years (range 60–81) and the median number of cycles delivered was 2 (range 1–4). Of 13 evaluable patients, in 8 (61%), the administration of bortezomib resulted in an antileukemic effect as demonstrated by peripheral blood and/or bone marrow blast reduction. In 4 (50%) of these 8, a decrease by 37% of transfusion requirement was also observed (*P = *0.009). Overall median survival was 4 months (range 0.25–10). Neurotoxicity was the most frequent adverse event with 7 of 13 (54%) patients experiencing grades 3-4 peripheral neuropathy. Neurotoxicity led to treatment discontinuation in 4 (57%) of 7. In conclusion, the observed anti-leukemic activity of bortezomib indicates that there is room for designing additional studies in which combination with other chemotherapeutic agents should be considered. Clinical registration no.: EUDRACT 2006-006923-38.

## 1. Introduction

In spite of significant therapeutic improvements, a large proportion of patients affected with acute myeloid leukemia (AML) relapse or are primary refractory to treatment [1]. In this population, comorbidities and poor performance status (PS) often preclude administration of additional intensive treatments. For the same reasons, more than 50% of elderly with previously untreated AML are considered not eligible for intensive therapy [2]. Overall, these categories of patients have a very unfavorable prognosis and demand for alternative strategies is insistent. Among novel agents, bortezomib is a dipeptidyl boronic acid with a potent and selective proteasome inhibitory activity. Bortezomib has demonstrated significant activity against a wide spectrum of human cancer cells [3] as well as a number of plasma cell lines and primary cells from patients with multiple myeloma, non-Hodgkin lymphoma, and chronic lymphocytic leukemia, and it has nowadays become a pivotal drug in the treatment of multiple myeloma [4]. The previous hypothesis was that bortezomib, by proteasome inhibition, inhibits IkB*α* degradation that in turn switches off NF-*κ*B pathway. NF-*κ*B is a key transcription factor constitutively activated in neoplastic cells from solid tumors and hematological malignancies. More recent evidences demonstrate that bortezomib shows an important role in disrupting a network that operates on the basis of interactions of miR-29b, the transcription factor SP1, and NF-*κ*B (p65). This network affects the expression of several genes in AML, including DNA methyltransferase enzymes and the receptor tyrosine kinase KIT [5]. These results supported the notion that miR-29b/SP1/NF-*κ*B(p65) complex-dependent KIT overexpression contributes to the growth of leukemia and could be targeted by bortezomib [5, 6]. Accordingly, attempts have been made to transpose the experimental data into the clinical scenario of AML treatment. A phase I study of bortezomib in patients with refractory/relapsed acute leukemia found that the maximum tolerated dose was 1.25 mg/m^2^. At this level, hematological evidence of antileukemic activity was observed. In a further study, higher doses of bortezomib (1.5 mg/m^2^) were administered in combination with idarubicin and cytarabine [7] without evidence of additional toxicity. In a very recent trial, escalating doses of bortezomib (0.7 to 1.3 mg/m^2^) were associated with decitabine for the treatment of high-risk AML patients [8]. The authors reported an overall response rate of 37% without significant toxicities. In line with these therapeutic efforts, we planned to test the clinical activity of bortezomib as single agent at the dose of 1.5 mg/m^2^ in 14 patients with untreated or relapsed/refractory AML, unfit for standard chemotherapy.

## 2. Patients and Methods

### 2.1. Patients Population

All patients were ≥18 years of age and were required to read and sign an informed consent document. Approval for this study was obtained from the institutional review board. Informed consent was obtained from patients in accordance with the declaration of Helsinki. Patients were eligible for enrollment if they had untreated or relapsed/refractory AML, unfit for conventional chemotherapy. The clinical characteristics of patients and the inclusion/exclusion criteria are summarized in Tables 1 and 2, respectively.

### 2.2. Treatment Schedule

Bortezomib was given as single agent at the dose of 1.5 mg/m^2^ twice weekly for two weeks of a 21-day cycle, for a maximum of 8 cycles.

### 2.3. Response Definition

Complete remission (CR) was defined as platelet count >100  ×  10^9^/L, neutrophil count (ANC) >1  ×  10^9^/L, and a cellular marrow with blast count <5%. Partial remission (PR) was defined as CR but with a decrease of at least 50% in the percentage of blasts to 5–25% in the bone marrow (BM) aspirate [1]. Treatment failure was arbitrarily defined as a progressive increase of the circulating and/or BM blasts, persisting in spite of at least 1 cycle administration. Disease conditions not complying with the criteria of any of these categories were classified as stable disease. Treatment was stopped if any of the following events occurred: treatment failure, concurrent illness that prevented further administration of treatment, unacceptable toxicity, or patient's consent withdrawal.

### 2.4. Safety and Efficacy Monitoring

BM aspirate was performed baseline and on day 1 of each cycle and at the final visit. Hematology and chemistry tests were performed baseline and on days 4, 8, and 11 of each cycle. The study also included safety and tolerability analysis. Adverse events have been reported from the initiation visit until the final visit and subsequent followups. Adverse events were reported according to the *National Cancer Institute Common Toxicity Criteria (NCI-CTC) v. 3*, and relationship with study drug was also evaluated. In particular, peripheral sensory neuropathy was graded as follows: Grade 1—asymptomatic, loss of deep tendon reflexes or paresthesia; Grade 2—moderate symptoms limiting activities of daily living (ADL); Grade 3—severe symptoms limiting self-care ADL; Grade 4—life-threatening consequences with urgent intervention indicated.

### 2.5. Cytogenetic Analysis

A G-banded chromosome study was performed on diagnostic bone marrow (BM) samples using standard cytogenetic techniques. Briefly, two unstimulated cultures were started in RPMI 1640 medium enriched with 20% fetal calf serum, L-glutamine, and antibiotics (penicillin and streptomycin). The cells were cultured for 24 and 48 hours in a 37°C incubator until harvest. Before harvesting, the cultures were treated with Colcemid (0.05 mg/mL) for 16–18 hours. Soon after, the cells were exposed to hypotonic solution (0.075 mol/L KCl) and fixed with methanol/acetic acid (3 : 1). Slides of the cells were prepared and stained using a G-banding (Trypsin-Giemsa-Wright) technique. Karyotyping was carried out on GTG-banded chromosomes and reported using the ISCN-1995 nomenclature, after analyzing a minimum of 20 metaphases for cases with no clonal aberrations [9]. 

### 2.6. Outcome Definition and Statistical Analysis

Comparisons between groups were made using the Chi-squared test or Fisher's exact test for categorical data. Overall survival (OS) was calculated from the date of entry into the trial to the date of death from any causes or last followup. Probabilities of OS were calculated using the Kaplan-Meier method. Univariate comparisons were made using the log-rank test for survival parameters.

## 3. Results

**Figure 1:**
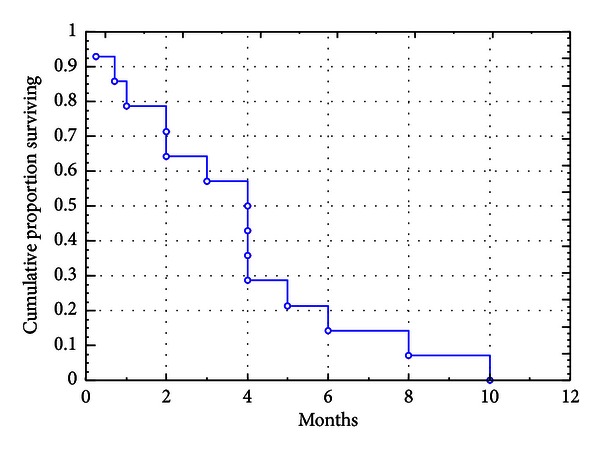
Kaplan-Maier curve of survival in the overall study group. Overall median survival was 4 months (range 0.25–10 months).

The clinical characteristics of the 14 patients enrolled into the protocol are summarized in Table 1. Median age was 70 (range 60–81), 5 patients had a previously untreated AML, 4 had refractory, and 5 relapsed AML. At the time of clinical evaluation, all 14 patients were considered ineligible for standard, intensive chemotherapy and therefore recruited to the bortezomib clinical trial. As for as patients with de novo AML, 3 patients have been considered frail due to age higher than 75 years, one refused conventional chemotherapy, and two had secondary leukemia following myelodysplasia being not eligible for conventional protocols available at that moment. The median number of cycles of bortezomib delivered was 2 (range 1–4). One patient died within 7 days from bortezomib initiation because of pulmonary infection, and therefore he was considered not evaluable for response. Although no instances of CR or PR were documented, 8 (61%) of 13 evaluable patients exhibited some degree of anti-leukemic effect generated by bortezomib (Table 3). Such an anti-leukemic action consisted in a median decrease by 53% (range 37%–99%) of peripheral blast count in 7 patients, and by 10% (range 6%–60%) of BM blasts in 4. In 3 patients, peripheral blast count and BM blast reduction were concurrent whereas in 4 and 1, an isolated peripheral blast count and BM blast reduction were recorded, respectively (Table 3). Moreover, in 4 (50%) of the 8 patients showing signs of anti-leukemic activity of bortezomib, a decrease by 37% of transfusion requirement was observed (mean 2.37 PRC/wk versus 0.87 PRC/wk; *P* = 0.009). In 8 patients showing anti-leukemic activity, the median number of cycles administered was 3 (range 1–4); treatment was withdrawn because of disease progression (4), toxicity (3), and patient refusal (1). The only patient with a stable disease, even though not experiencing any peripheral blood or BM blast improvement, did attain an increase of ANC (Table 3) and eventually progressed. All the 8 patients in whom bortezomib induced an anti-leukemic effect and the one with stable disease had no unfavorable karyotype or peripheral blast count ≥5  ×  10^9^/L at the time of entry into the study. On the other hand, among the 4 patients who progressed, 2 carried unfavorable karyotype (chromosome 7 monosomy and complex karyotype) and 2 had a peripheral blast count > 5  ×  10^9^/L. Median overall survival for the whole group was 4 months (range 0.25–10) (Figure 1).

Four patients (29%) developed grade 3 febrile neutropenia and 5 pulmonary infection that was fatal in 4; in 2 of these 4, pneumonitis occurred during AML progression. Seven patients out of 13 (54%) experienced a peripheral sensory neuropathy (3 grade 1; 1 grade 2; 1 grade 3; 2 grade 4) with all of them having a previous history of chemotherapy. Among the 2 patients who developed grade 4 peripheral neuropathy, 1 suffered from a mixed sensory motor axonal polyneuropathy involving upper and lower limbs and causing impairment in walking and bladder palsy. The other one experienced sensory motor polyneuropathy and anal incontinence that resolved after one month following drug withdrawal. Neurological toxicity led to treatment discontinuation in 4 (57%) patients. No differential incidence of adverse events has been observed between the 8 patients showing antileukemic activity as compared to the others.

## 4. Discussion

Despite the advances in the treatment of AML, relapse after a chemotherapy-induced remission or resistance to front-line therapy still represents a critical issue since patients experiencing such conditions have a very dismal outlook. In this context, elderly patients represent a category at very high risk not only due to the frequent inability to afford intensive chemotherapy but also to the few chances of prolonged remission with standard chemotherapy. Based on this, efforts are under way to implement the use of novel agents with the aim to target leukemic cells while sparing normal ones from the broad attack of chemotherapy or, alternatively, that can be safely combined with chemotherapy. Bortezomib is a potent proteasome inhibitor that has been demonstrated to be very effective in multiple myeloma [10]. Although not fully elucidated, the cytotoxic effects of bortezomib might be related to the indirect inhibition of several target genes that are critical to AML growth [5–11]. In vitro, bortezomib has also been found to inhibit AML blast survival and to interfere with the interaction between AML progenitors and microenviromental niche [12]. Clinical experience of bortezomib in AML is limited to few reports. Cortes et al. [7] treated 15 relapsed/refractory AML patients with escalating doses of bortezomib. Dose-limiting toxicity was identified at 1.5 mg/m^2^ and included orthostatic hypotension, nausea, diarrhea, and fluid retention; therefore, the maximum tolerated dose was set at 1.25 mg/m^2^. Clinical efficacy consisted in hematological improvement observed in 5 (33%) patients. In a further experience, Attar et al. [13] treated 31 patients with high-risk AML by associating bortezomib at the dose of 1.5 mg/m^2^ with cytarabine and idarubicin. They reported a CR rate of 61% with an acceptable and manageable toxicity consisting in hypoxia, hyperbilirubinemia, and elevated aspartate aminotransferase. Blum et al. [8] treated 19 high-risk AML patients with a combination of escalating doses of bortezomib (0.7–1.3 mg/m^2^) and decitabine, achieving an overall response rate of 37% with mild additional toxicity. Furthermore, Attar et al. published the results of 98 patients treated with bortezomib in combination with daunorubicin and cytarabine with a CR frequency of 65% [14]. Lancet et al. treated 27 patients (26 AML and 1 ALL patients) with tipifarnib and bortezomib with escalation doses of both drugs. CR with incomplete count recovery was observed in 2 patients (7%), and additional 5 patients (19%) had stable disease [15].

**Figure 2:**
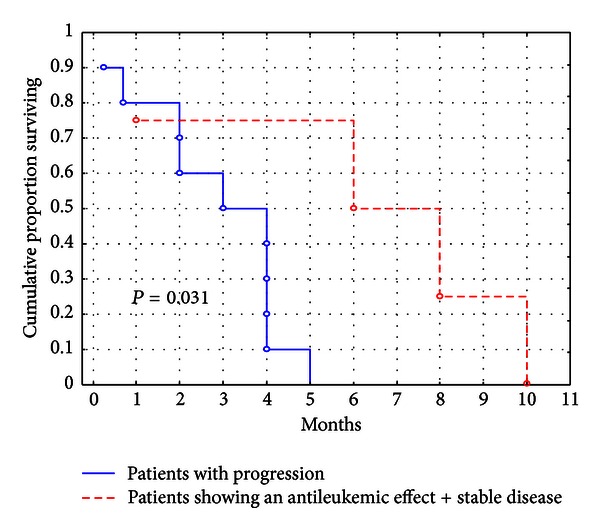
Survival of patients achieving an antileukemic effect or stable response compared with that of patients showing disease progression. Patients for whom an anti-leukemic effect was observed had a mean duration of survival that doubled the one of those with a bortezomib fully insensitive disease (4 months versus 2 months; *P* = 0.031).

We designed a phase 2 trial of bortezomib single-agent delivered at the dose of 1.5 mg/m^2^, injected twice weekly for two weeks of a 21-day cycle, for a maximum of 8 cycles. We recruited to the trial a series of 14 patients bearing a very high-risk profile due to age, relapse/refractory disease, and poor performance status. Even though instances of CR or PR were not observed, in 61% of evaluable patients, bortezomib produced some degree of anti-leukemic effect consisting in peripheral and/or BM blast decrease that in 3 cases was concurrent. The median OS for the whole population was 4 months (Figure 1); however, patients for whom an anti-leukemic effect was observed had a duration of survival (Figure 2) that doubled the one of those with a bortezomib fully insensitive disease: 4 months (range 3–8) versus 2 months (range 1-2); (*P* = 0.031). This is in line with the assumption that, at least in the setting of experimental therapies, also “less than CR” responses may contribute to prolong survival. Due to the limited numbers in our series, we were not able to accomplish any subanalysis; however, unfavorable karyotype and high peripheral blast count were more frequently associated with a status of bortezomib full-insensitivity. Neurologic toxicity was the most frequent and worrisome side-effect with a significant proportion of our patients suffering from peripheral neuropathy, leading to treatment discontinuation in 4. Cortes et al. [7] reported that, at the dose of 1.5 mg/m^2^, 2 of 5 patients developed neuropathy (both grade <3). Blum et al. [8] described instances of grades 3-4 neuropathy in 3 patients after repetitive cycles. Differently, in the first report by Attar et al. [13], no cases of neuropathy were observed. In the updated one, 11 patients developed grade 3 sensory neuropathy during treatment course, 6 during the first induction cycle, 3 during the second induction cycle, and 2 during consolidation. The neuropathy resolved within weeks of treatment [14]. Lancet et al. described 1 additional case of grade 3 sensorimotor neuropathy [15]. Since our 7 patients experiencing neuropathy have been already exposed to chemotherapy, we speculated that previous treatments might have made them more prone to develop neurologic complications while on bortezomib. In fact, patients treated with frontline bortezomib developed only grade 0/1 peripheral neuropathy.

In conclusion, in our experience, bortezomib showed a limited but clear anti-leukemic effect; a caveat is represented by the peripheral nerve status that should be carefully evaluated to avoid excessive toxicity and treatment withdrawal. Trials of combination with other anti-leukemic agents are warranted to explore potential synergistic effects between bortezomib and other cytotoxic drugs.

## Figures and Tables

**Table 1:** Clinicobiological characteristics of the patients.

	Patients No. (%)
Age	
>70	7 (50)
<70	7 (50)
Sex	
Male	10 (71)
Female	4 (29)
FAB	
M4	8 (57)
M5	4 (29)
M6	2 (14)
Disease status	
De novo	3 (21)*
Secondary	2 (14)**
Refractory	4 (29)
Relapsed	5 (36)
Previous lines of CHT	
1	4
2	**1**
>2	4
Peripheral blood blast count	
<5 × 10^9^/L	9 (64)
>5 × 10^9^/L	5 (36)
Karyotype	
Intermediate	10 (83)
Unfavorable	2 (17)
Failure	2

*Frails due to age higher than 75 years, one refused conventional chemotherapy.

**Secondary leukemia following myelodysplasia and not eligible for the available conventional protocols.

**Table 2:** Inclusion and exclusion criteria.

Inclusion criteria	Exclusion criteria
Patients with primary or secondary AML, either de novo or relapsed/refractory, considered not candidates for conventional chemotherapy	Candidate for allogeneic bone marrow transplantation
Karnofsky Performance status > 60%	Presence of central nervous system leukemia or any medical psychiatric condition
Adequate hepatic and renal function	Active uncontrolled bacterial infection or HIV infection
Age 18 years or greater	Uncontrolled diabetes and severe cardiovascular disease (NYHA)
Documentation if written informed consent	History of hypotension
At least 4 weeks from prior chemotherapy	Pregnant or breastfeeding
Either men or women, accepting to practice effective contraception during the entire study period	Neuropathy > grade 2 and receipt of extensive radiation therapy, systemic chemotherapy.

**Table 3:** The extent of antileukemic effect induced by bortezomib is reported for peripheral blood and bone marrow of 13 evaluable patients affected with acute myeloid leukemia. Comparison was performed between baseline values and the time when the maximum effect was recorded.

Patients	Maximum variation in PB blast count (×10^9^/L)	Maximum variation in BM blast (%)	Variation in ANC (×10^9^/L)	Therapy duration (days)	Antileukemic activity (% reduction)^1^
1	0.37→0.02	n.v.	0.15→0.32	11	↓PB (93%)
2	0.54→0.34	53→50	3.3→1.2	52	↓PB (37%), ↓BM (6%)
3	0.04→1	60→91	0.16→0.02	85	progression
4	1.81→1.1	40→16	0.98→0.25	32	↓PB (39%), ↓BM (60%)
5	7.22→3.42	n.v.	0→0.24	32	↓PB (53%)
6	1.73→1.80	70→70	1.01→1.78	28	stable disease
7	2.5→5.5	60→60	4.64→2.1	30	progression
8	0.95→1.8	45→80	0.74→1.78	30	progression
9	2.31→74.16	75→70	1.87→2.79	11	↓BM (7%)
10	1.2→0.02	30→58	0.51→1.0	54	↓PB (99%)
11	0.13→0.09	80→80	0.6→1	90	↓PB (93%)
12	0.36→1.27	17→50	0.35→0.35	58	progression
13	1.2→0.8	40→35	1.8→1.8	11	↓PB (33%) ↓BM (13%)

Abbreviations: PB: peripheral blood; BM: bone marrow; ANC: absolute neutrophil count; n.v.: not evaluable; ↓PB: decrease in PB blast count; ↓BM: decrease in BM blast count.

^1^In brackets, PB and/or BM blast reduction is reported as percentage difference compared to the baseline evaluation.
